# Exploring the Antibacterial, Anti-Inflammatory, and Antioxidant Properties of the Natural Food Supplement “*Protegol*” as a Supportive Strategy in Respiratory Tract Infections

**DOI:** 10.3390/antibiotics14121260

**Published:** 2025-12-13

**Authors:** Alexia Barbarossa, Maria Pia Argentieri, Maria Valeria Diella, Eleonora Spinozzi, Filippo Maggi, Antonio Carrieri, Filomena Corbo, Antonio Rosato, Alessia Carocci

**Affiliations:** 1 Department of Pharmacy-Pharmaceutical Sciences, University of Bari “Aldo Moro”, Via E. Orabona, 4, 70125 Bari, Italy; mariapia.argentieri@uniba.it (M.P.A.); m.diella2@phd.uniba.it (M.V.D.); antonio.carrieri@uniba.it (A.C.); filomena.corbo@uniba.it (F.C.); antonio.rosato@uniba.it (A.R.); 2 Chemistry Interdisciplinary Project (ChIP) Research Center, School of Pharmacy, University of Camerino, Via Madonna delle Carceri, 62032 Camerino, Italy; eleonora.spinozzi@unicam.it (E.S.); filippo.maggi@unicam.it (F.M.)

**Keywords:** antibacterial activity, respiratory infections, bioactive compounds

## Abstract

**Background/Objectives**: Respiratory tract infections (RTIs) remain a leading cause of morbidity worldwide and are frequently associated with the emergence of multidrug-resistant pathogens. In this context, natural compounds represent a valuable source of novel antimicrobial and immunomodulatory agents. The present study aimed to evaluate the antibacterial, anti-inflammatory, and antioxidant activities of *Protegol*, a natural food supplement enriched in bioactive phytochemicals including hydroalcoholic extracts of propolis and hedge mustard (*Sisymbrium officinale* (L.) Scop.) aerial parts, together with honey, against clinically relevant bacterial strains and in cellular models of inflammation and oxidative stress. Furthermore, the ability of the multi-herbal formulation to alter the permeability of the bacterial cell wall was assessed. **Methods**: The antibacterial properties of *Protegol* were evaluated by determining its minimum inhibitory (MIC) and minimum bactericidal concentrations (MBC) against a panel of Gram-positive and Gram-negative bacteria, using the broth microdilution method. Cell wall permeability was investigated through the propidium iodide (PI) uptake assay. The anti-inflammatory potential was investigated in LPS-stimulated RAW 264.7 macrophages by measuring nitric oxide (NO) production with the Griess assay. The antioxidant activity was evaluated in BALB/3T3 fibroblasts exposed to hydrogen peroxide, using the DCFH-DA assay. **Results**: *Protegol* exhibited a broad-spectrum antibacterial effect, with MIC values ranging from 1.5 to 6.2 mg/mL and MBC values between 3.1 and 12.4 mg/mL. The strongest activity was observed against *Staphylococcus aureus* and *Streptococcus pyogenes*, including clinical isolates, while moderate efficacy was detected against resistant *Klebsiella pneumoniae* strains. PI uptake assays confirmed a dose-dependent disruption of bacterial membrane integrity, supporting a direct effect of *Protegol* on cell wall permeability. In macrophages, *Protegol* significantly and dose-dependently reduced NO release, lowering production to 44% at the highest concentration tested. In BALB/3T3 cells, *Protegol* markedly decreased ROS accumulation to 24% at the same concentration. **Conclusions**: Overall, the findings support the potential of *Protegol* as a natural adjuvant to the conventional therapies for respiratory tract health by counteracting bacterial pathogens, reducing inflammation, and mitigating oxidative stress, thereby supporting host defense mechanisms in the context of respiratory tract infections.

## 1. Introduction

Antibiotic resistance remains one of the most pressing global health challenges of the 21st century, undermining the effectiveness of standard antimicrobial therapies and contributing substantially to higher global rates of morbidity, mortality, and healthcare-related costs [1]. The rapid emergence and dissemination of resistant bacterial strains have rendered many first-line antibiotics less effective, also in the treatment of common infectious diseases, prompting an urgent need for alternative or complementary therapeutic strategies [2]. Among the various infectious diseases impacted by antimicrobial resistance, respiratory tract infections (RTIs) stand out due to their global prevalence and clinical impact. Lower respiratory tract infections (LRTIs), such as pneumonia and bronchiolitis, remain a leading cause of death and disability, affecting nearly 500 million people and accounting for approximately 2.4 million deaths annually [3]. These infections affect individuals across all age groups but pose the greatest threat to vulnerable populations such as children under five years of age and the elderly. Key pathogens implicated in severe RTIs include *Staphylococcus aureus*, *Klebsiella pneumoniae*, *Streptococcus pyogenes*, and *Pseudomonas aeruginosa*, particularly in hospitalized or immunocompromised patients [4].

Of particular concern are *Streptococcus*-related respiratory infections, which represent some of the most frequent infections in pediatric populations [5]. These pathogens are responsible for a wide range of clinical manifestations, including otitis media, sinusitis, pharyngitis, and pneumonia [6]. In certain cases, streptococcal infections may also lead to serious complications such as rheumatic fever, meningitis, or post-streptococcal glomerulonephritis [7]. Alarmingly, approximately 20% of *Streptococcus* isolates in pediatric patients across Europe exhibit resistance to at least one first-line antibiotic, further compounding the clinical burden and reducing available treatment options [8].

Given the growing threat of antibiotic resistance and the limitations of conventional treatments, there is a clear and urgent need to explore novel therapeutic approaches or adjuvant strategies that can support or enhance current interventions, particularly in the context of RTIs [9]. In this regard, natural compounds have emerged as promising candidates due to their multi-target activities. Natural molecules and plant-derived extracts are increasingly recognized for combining antimicrobial, redox-balancing, and inflammation-modulating effects. These natural products typically act through multiple biological targets, and their combination can result in synergistic enhancement of overall effectiveness. Increasing attention has been devoted to multi-herbal formulations for their capacity to influence molecular networks governing infection defense, immune homeostasis, and tissue repair [10]. A practical and widely adopted approach for delivering these bioactive molecules is the use of nutraceuticals or dietary supplements, which often include standardized extracts to guarantee uniform levels of active components and exhibit good tolerability [11]. Oral administration ensures ease of use and facilitates daily intake, supporting their use as accessible tools to deliver active compounds that may help reduce microbial burden, attenuate inflammation, counteract oxidative stress, and contribute to mucosal protection in the respiratory tract [12]. Only a limited number of investigations have thoroughly evaluated the biological properties of commercial supplements within models of respiratory infections [13]. The dietary supplement investigated in this study is *Protegol*, a multi-component formulation specifically developed for respiratory well-being. Its composition integrates propolis, honey, medicinal plant extracts, micronutrients, and essential oils, traditionally employed for their antimicrobial, anti-inflammatory, and immunomodulatory properties.

Propolis is a resinous substance collected by bees from plant exudates, rich in flavonoids, phenolic acids, and their esters. Among its most studied constituents, caffeic acid phenethyl ester (CAPE), galangin, and pinocembrin have been shown to exert broad-spectrum antibacterial, antioxidant, and anti-inflammatory activities, contributing to mucosal protection and immune modulation [14]. Honey, a natural matrix composed of sugars, enzymes, polyphenols, and trace elements, complements the action of propolis by exerting antimicrobial and soothing effects on the upper respiratory tract, partly attributed to hydrogen peroxide release and bioactive phytochemicals [15]. The formulation also includes *Sisymbrium officinale* (L.) (hedge mustard), another component comprising the formulation that is traditionally known as “singer’s plant” for its soothing action on the oropharyngeal mucosa. Its aerial parts contain glucosinolates and sulfur compounds, which are believed to contribute to anti-inflammatory and expectorant properties, supporting its ethnopharmacological use in hoarseness, pharyngitis, and laryngitis [16]. In addition, *Protegol* is enriched with vitamin C (L-ascorbic acid) and zinc gluconate, two micronutrients with well-established roles in respiratory defense. Vitamin C functions as a potent antioxidant and immune-supporting molecule, protecting epithelial barriers and reducing ROS levels [17], whereas zinc is essential for mucosal immunity and exhibits antiviral and antibacterial properties [18].

Finally, the blend of essential oils provides additional antimicrobial and anti-inflammatory components. *Leptospermum scoparium* J.R. Forst. & Forst. (*manuka* oil) is generally rich in β-triketones, such as leptospermone, with potent activity against Gram-positive bacteria [19]. *Eucalyptus globulus* Labill. oil, characterized by its high content of 1,8-cineole, has been widely documented for its mucolytic, antimicrobial, and anti-inflammatory effects in respiratory conditions [20]. *Melaleuca alternifolia* (tea tree oil), containing terpinen-4-ol and γ-terpinene, exerts broad-spectrum antibacterial and antifungal activity and has been investigated for its efficacy in respiratory and mucosal infections [21,22]. The purpose of this study was to explore the biological properties of *Protegol*, a formulation combining natural ingredients beneficial for respiratory tract health. Specifically, we investigated its in vitro antibacterial activity against clinically relevant respiratory pathogens, including both reference and multidrug-resistant strains, together with its effects on bacterial membrane permeability, NO production in macrophages, and ROS modulation in fibroblasts. Taken together, these experiments aim to provide deeper insights into the in vitro biological profile of *Protegol*.

## 2. Results

### 2.1. Protegol Chemical Composition

To study the chemical composition of *Protegol*, the commercial product was divided into two fractions—polar and apolar—based on the ingredients listed on the packaging. Hexane was added to the commercial product, causing phase separation. The upper phase, containing hexane, is referred to as the apolar fraction and is rich in essential oils. The lower phase, containing water, is referred to as the polar fraction. The HPLC-DAD analysis of the polar fraction indicated a very complex composition. The MS and MS/MS spectra of the polar fraction enable the identification of several compounds, including caffeic acid, p-coumaric acid, ferulic acid, isoferulic acid, and 3,4-dimethylcaffeic acid (DMCA). Four caffeic acid derivatives were also identified in the analyzed samples, including caffeic acid prenyl ester, caffeic acid benzyl ester, and caffeic acid phenylethyl ester (CAPE). Table S1 (Supplementary Materials) presents the UV and mass spectral data for each compound identified. Certain compounds were confirmed by direct comparison with authentic reference standards, whereas others were tentatively assigned through UV and MS spectral interpretation supported by literature data [23]. The combined application of HPLC-PDA and LC-MS analyses (Table S1, Supplementary Materials) verified the presence of both flavonoids and phenolic acids in *Protegol*. Quantitative analysis revealed that flavonoids were the predominant constituents of *Protegol*, accounting for approximately 64.5% of the total identified compounds, with chrysin and pinocembrin as the major representatives. Phenolic acids comprised about 35% of the composition, among which caffeic acid phenylethyl ester (CAPE) was the most abundant. About the EO characterization, as expected, 1,8 cineol and terpin-4-ol were predominant because of they were the identification markers of *Eucaliptus globulus* essential oil and of *Melaleuca alternifolia* essential oil, respectively [21]. Table S2 (Supplementary Materials) showed the completed characterization of the apolar fraction.

### 2.2. Antibacterial Effects of Protegol

The antibacterial activity of *Protegol* was evaluated according to the Clinical and Laboratory Standards Institute (CLSI) guidelines (document M07-A9, 2012) [24]. The bacterial panel included a range of clinically relevant Gram-positive and Gram-negative strains, comprising both American Type Culture Collection (ATCC, Manassas, VA, USA) reference strains and clinical isolates (Table 1). The selection of these microorganisms was guided by their well-documented role in respiratory tract infections (RTIs), including pneumonia, pharyngitis, and otitis media, conditions that remain among the leading causes of morbidity worldwide. Several of the selected pathogens, including *Streptococcus pneumoniae*, *Staphylococcus aureus* (including MRSA), *Klebsiella pneumoniae* and *Pseudomonas aeruginosa*, are recognized for their ability to develop multidrug resistance, further compounding the challenge of effective treatment. *Protegol* exhibited a broad-spectrum inhibitory effect, with MIC values ranging from 1.5 to 6.2 mg/mL and MBC values between 3.1 and 12.4 mg/mL. Among Gram-positive strains, *S. aureus* ATCC 25923 and ATCC 29213 were the most susceptible, both inhibited at 1.5 mg/mL with bactericidal activity at 3.1 mg/mL. The MRSA strain *S. aureus* ATCC 43300 required slightly higher concentrations, with inhibition achieved at 3.1 mg/mL and bactericidal activity at 6.2 mg/mL, although it remained within the effective range. Regarding *Streptococcus* spp., *Protegol* showed variable but significant activity. *S. pyogenes* ATCC 19615 was inhibited at 3.1 mg/mL with a bactericidal effect at 6.2 mg/mL, with an higher sensitivity observed for the corresponding clinical isolate *S. pyogenes* F, which was inhibited at 1.5 mg/mL and eradicated at 3.1 mg/mL. This strong activity is of particular interest, as *S. pyogenes* is a primary etiological agent of upper respiratory tract infections, such as pharyngitis and tonsillitis, and can cause severe invasive diseases. *S. mutans* ATCC 25175 and *S. sanguinis* ATCC 10556 were also inhibited at 3.1 mg/mL and killed at 6.2 mg/mL, suggesting that *Protegol* may interfere with oral streptococci that, although generally commensal, can act as opportunistic pathogens in respiratory or systemic infections. *S. pneumoniae* ATCC 10015 and *S. salivarius* ATCC 13419 were less susceptible, requiring 6.2 mg/mL for inhibition and 12.4 mg/mL for bactericidal activity. Concerning Gram-negative bacteria, *K. pneumoniae* ATCC 13883 was inhibited at 3.1 mg/mL and eradicated at 6.2 mg/mL, while the resistant strain *K. pneumoniae* ATCC 700603 and the clinical isolate required higher concentrations, with inhibition achieved at 6.2 mg/mL and bactericidal activity at 12.4 mg/mL. A similar profile was observed for *P. aeruginosa* ATCC 27853, which displayed inhibition at 6.2 mg/mL and complete eradication at 12.4 mg/mL, confirming the slightly lower susceptibility of this pathogen, consistent with its well-known intrinsic resistance mechanisms. To validate the assay, levofloxacin, a well-known reference antibiotic, was included as a positive control, and the MIC values obtained were consistent with the susceptibility ranges reported by CLSI.

### 2.3. Evaluation of Bacterial Cell Wall Permeability of Protegol

**Figure 1:**
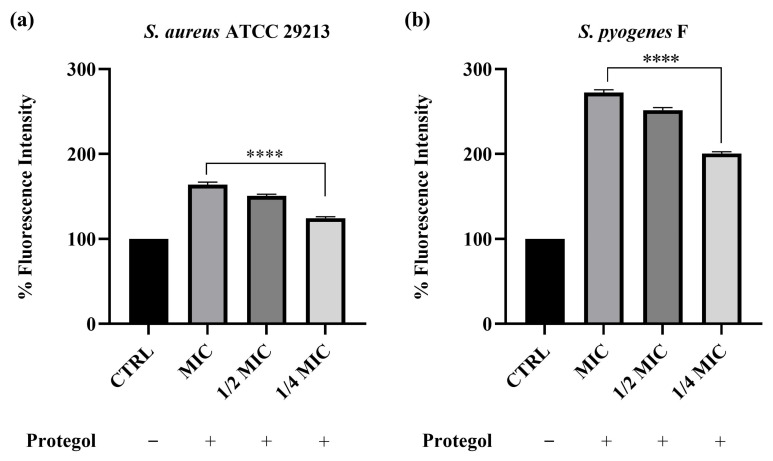
Cell wall permeability of *Staphylococcus aureus* ATCC 29213 (**a**) and *Streptococcus pyogenes* F (**b**) upon incubation with *Protegol* at MIC, ½ MIC, and ¼ MIC concentrations. “+” and “−” denote treated and untreated samples, respectively. Fluorescence intensity is expressed as a percentage relative to the untreated control (CTRL, set at 100%). Data are presented as mean ± standard deviation (SD) of three independent experiments (*n* = 3). Significant differences versus CTRL: **** *p* < 0.0001. Results are shown as mean ± standard deviation (SD) (*n* = 3).

To investigate whether the antibacterial effect of *Protegol* was associated with alterations in bacterial membrane integrity, the propidium iodide (PI) uptake assay was carried out. This test relies on the ability of PI to enter cells only when the membrane is compromised, where it binds nucleic acids and emits a strong fluorescent signal, thus providing a direct indication of increased permeability. The assay was carried out on *Staphylococcus aureus* ATCC 29213 and the clinical isolate *Streptococcus pyogenes* F, which were selected from the bacterial panel based on their higher susceptibility to *Protegol* in MIC/MBC assays. As shown in Figure 1, treatment with *Protegol* caused a clear and concentration-dependent increase in PI uptake in both *Staphylococcus aureus* ATCC 29213 and the clinical isolate *Streptococcus pyogenes* F compared to untreated controls (set at 100%). In *S. aureus*, fluorescence values increased to 163% at the MIC value, 150% at ½ MIC, and 124% at ¼ MIC (Figure 1a), confirming that even sub-inhibitory concentrations were able to partially compromise cell wall integrity. The effect was even more pronounced in *S. pyogenes*, where PI uptake rose dramatically to 272% at MIC, 251% at ½ MIC, and 200% at ¼ MIC (Figure 1b). These findings demonstrate that *Protegol* strongly enhances bacterial membrane permeability, with a more marked effect on *S. pyogenes* than on *S. aureus*.

### 2.4. Anti-Inflammatory Activity of Protegol Against LPS-Induced Nitric Oxide Production

**Figure 2:**
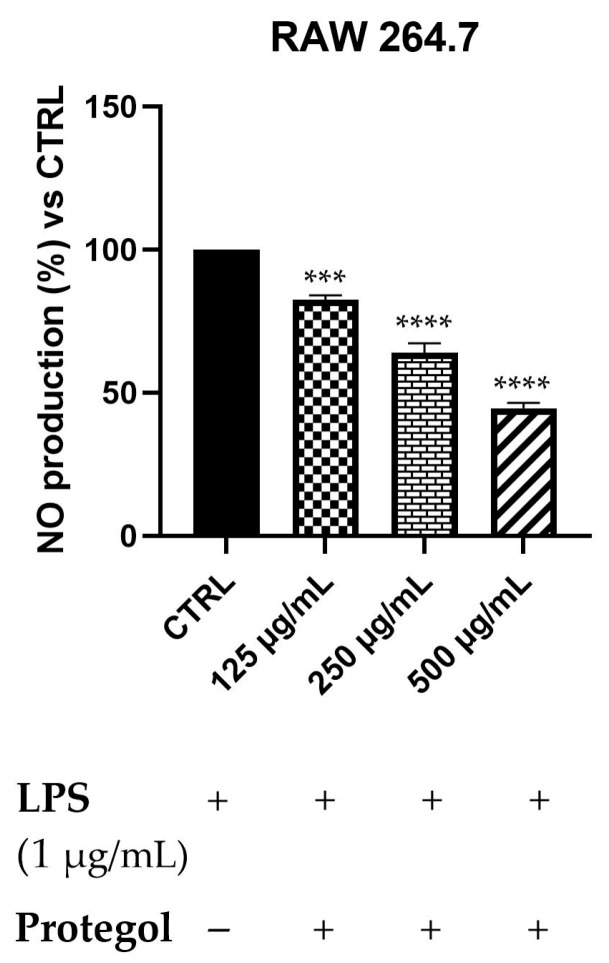
Nitric oxide (NO) levels in RAW 264.7 macrophages stimulated with LPS (1 μg/mL) and treated with increasing concentrations of *Protegol* (125, 250, and 500 μg/mL). “+” and “−” denote treated and untreated samples, respectively. NO production was quantified using the Griess assay and expressed as a percentage relative to the LPS-treated control. Data are presented as mean ± standard deviation (SD) of three independent experiments (*n* = 3). Significant differences versus CTRL: *** *p* < 0.001, and **** *p* < 0.0001.

To assess the potential anti-inflammatory effects of *Protegol*, the NO production in LPS-stimulated RAW 264.7 macrophages has been measured using the Griess assay. An initial MTT viability test was carried out to rule out any cytotoxic effects of *Protegol*. The results showed no significant decrease in cell viability even at the maximum concentration tested (500 μg/mL), supporting the suitability of these doses for subsequent analyses. As illustrated in Figure 2, exposure to *Protegol* at 125 μg/mL led to a significant reduction in NO release, reaching 82% of the value observed in LPS-stimulated controls. Increasing the concentration to 250 μg/mL and 500 μg/mL further lowered NO levels to 64% and 45%, respectively. These results demonstrate a marked, concentration-dependent suppression of NO synthesis, highlighting the pronounced anti-inflammatory activity of *Protegol* in LPS-activated macrophages.

### 2.5. ROS Scavenging Effects of Protegol Against H_2_O_2_-Induced Oxidative Stress

**Figure 3:**
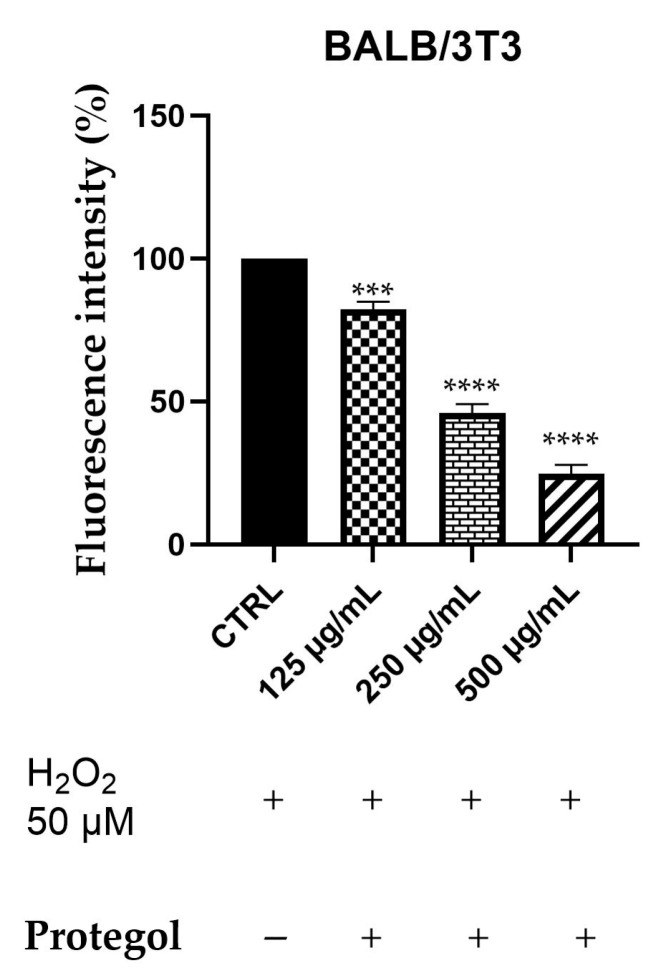
DCFH oxidation in BALB/3T3 cells after exposure to H_2_O_2_, 50 μM, and different concentrations of *Protegol* (125–500 μg/mL). “+” and “−” denote treated and untreated samples, respectively. The results are shown as mean ± standard deviation (SD) (*n* = 3). Significant differences versus CTRL: *** *p* < 0.001, and **** *p* < 0.0001.

Intracellular ROS generation was assessed in BALB/3T3 cells exposed to 50 μM H_2_O_2_ and treated with *Protegol* at 125, 250, and 500 μg/mL using the DCFH-DA fluorescence assay. The BALB/3T3 murine fibroblast line was selected as a representative model for studying cytoprotective and antioxidant responses in non-tumorigenic cells. As reported for the previous assay, the same concentrations of *Protegol* were tested, and to ensure that they were not cytotoxic also in this cellular model, a preliminary MTT assay was performed, confirming that, also in this case, cell viability remained unaffected even at the maximum concentration tested (500 μg/mL), confirming the appropriateness of these doses for further assays. As depicted in Figure 3, *Protegol* treatment at 125 μg/mL lowered ROS levels to 82%, with a pronounced, dose-dependent decline to 45% and 24% observed at 250 μg/mL and 500 μg/mL, respectively.

## 3. Discussion

In recent years, the search for alternative strategies to prevent and manage respiratory tract infections (RTIs) has increasingly directed toward natural, low-toxicity agents that can complement or potentiate conventional treatments. pies [25]. RTIs, especially those caused by multidrug-resistant bacteria, remain a leading cause of morbidity and mortality worldwide, and the therapeutic arsenal is progressively threatened by the rise in antimicrobial resistance [26]. Current treatments are often limited to symptomatic relief or rely on antibiotics whose efficacy is increasingly compromised, underscoring the urgent need for new approaches that can provide both antimicrobial protection and modulation of host responses [27]. Natural products, particularly in the form of dietary supplements, have attracted growing interest due to their diverse phytochemical composition and multifunctional action on various biological pathways. Within this framework, multi-ingredient formulations designed to support respiratory health, such as *Protegol*, may offer a holistic approach by combining direct antibacterial effects with anti-inflammatory and antioxidant properties. The inclusion of propolis, honey, *Sisymbrium officinale* (L.), micronutrients, and essential oils (manuka, eucalyptus, tea tree) provides a complex phytochemical profile in which flavonoids, phenolic acids, terpenes, and vitamins can act in conjunction to reduce microbial burden, attenuate mucosal inflammation, and counteract oxidative stress. Comparable formulations have already been explored in the field of respiratory health, reinforcing the growing evidence that formulations combining propolis, honey, essential oils, and micronutrients can exert clinically relevant antimicrobial and immunomodulatory actions in the respiratory tract [28,29,30]. Against this background, our study aimed to further elucidate the biological activities of *Protegol*, integrating antibacterial, anti-inflammatory, and antioxidant assays to highlight its potential role as a supportive strategy in the complementary management of RTIs.

### 3.1. Antibacterial Effects of Protegol

The antibacterial activity observed for *Protegol* aligns with the existing literature on natural extracts and their bioactive constituents, underscoring the potential of propolis, honey, and essential oils, well-known for their antibacterial activities, as effective antimicrobial agents in the context of respiratory tract infections. *Protegol* displayed strong antibacterial effects against Gram-positive species, with *Staphylococcus aureus* being the most sensitive (MIC 1.5 mg/mL for methicillin-susceptible isolates and 3.1 mg/mL for MRSA). This potency is consistent with previous findings on propolis extracts, which demonstrated MICs ranging between 0.5 and 4.0 mg/mL against *S. aureus*, largely attributed to phenolic constituents such as CAPE, galangin, and pinocembrin [31]. Similarly, chlorogenic and caffeic acids—also present in propolis and honey—have been reported to inhibit *S. aureus* with MICs in the range of 0.6–10.0 mg/mL [10], values comparable to those observed for *Protegol*. The strong activity against *Streptococcus pyogenes* is of particular relevance, as this pathogen represents a leading cause of pharyngitis and tonsillitis. Both the ATCC strain and the clinical isolate demonstrated high sensitivity to *Protegol*, with MICs of 3.1 and 1.5 mg/mL, respectively. Comparable studies report MIC values in the same range for propolis and essential oils such as tea tree and manuka, whose major compound terpinen-4-ol is known to exert potent antibacterial effects against Gram-positive streptococci [32]. Additional inhibition of *S. mutans* and *S. sanguinis* suggests that *Protegol* may also impact commensal oral streptococci, which, under conditions of dysbiosis, can act as opportunistic pathogens in respiratory or systemic infections [33]. Conversely, *S. pneumoniae* and *S. salivarius* required higher concentrations (MIC 6.2 mg/mL), in line with previous evidence that pneumococci display lower susceptibility to natural extracts due to capsule-associated resistance mechanisms [34]. *Protegol* also demonstrated measurable activity against Gram-negative bacteria. The susceptibility of *Klebsiella pneumoniae* ATCC 13883 (MIC 3.1 mg/mL) is consistent with studies showing that propolis-derived phenolics and eucalyptus oil components such as 1,8-cineole compromise the integrity of the Gram-negative outer membrane and inhibit essential metabolic pathways [35]. The higher MIC values observed for the ESBL-producing strain (ATCC 700603) and the clinical isolate (6.2 mg/mL) reflect the intrinsic multidrug resistance and efflux mechanisms characteristic of these pathogens, which make them particularly challenging to treat. Although the MIC value was slightly higher, *Protegol* was nevertheless able to exert a notable inhibitory effect, an aspect of particular relevance in the case of resistant *K. pneumoniae*. It is noteworthy that, in this context, the detection of any antibacterial activity against resistant *K. pneumoniae*, reinforces the potential value of *Protegol* as an adjuvant in respiratory health [36]. Given that *K. pneumoniae* is poorly responsive to conventional therapies and, to the best of our knowledge, there are very few reports in the literature describing significant activity of natural formulations against this pathogen, the activity observed here represents a particularly remarkable finding. Overall, our findings confirm that the antibacterial effect of *Protegol* cannot be attributed to a single compound but instead is likely due to the concerted action of several bioactive constituents working in combination. This phytocomplex nature enhances its antimicrobial profile compared to individual components, particularly against Gram-positive pathogens such as *S. aureus* and *S. pyogenes*. In light of the increasing burden of antibiotic resistance, the demonstrated efficacy of *Protegol* against clinically relevant and resistant strains highlights its potential as a natural supplement to support respiratory tract health.

### 3.2. Evaluation of Bacterial Cell Wall Permeability of Protegol

The results of the propidium iodide (PI) uptake assay indicate that disruption of membrane integrity could be involved in the antibacterial activity of *Protegol*. The selection of *Staphylococcus aureus* ATCC 29213 and *Streptococcus pyogenes* F for this assay was based on their high susceptibility to *Protegol*, as these strains exhibited the lowest MIC values among all those tested. Focusing on strains with the strongest antibacterial response allows a more reliable assessment of potential membrane-targeting effects at sub-inhibitory concentrations and provides a clearer mechanistic readout. The marked increase in PI fluorescence observed in both *Staphylococcus aureus* ATCC 29213 and *Streptococcus pyogenes* F, particularly at MIC and sub-MIC concentrations, indicates that *Protegol* components are capable of compromising bacterial cell walls even at relatively low doses. This effect was especially pronounced in *S. pyogenes*, where PI uptake more than doubled compared to untreated controls, suggesting that this species may be intrinsically more vulnerable to membrane-targeting natural compounds. These results align with earlier reports on propolis and essential oils, where phenolic acids (e.g., caffeic and chlorogenic acid) and terpenes (such as 1,8-cineole and terpinen-4-ol) were shown to integrate into bacterial membranes, increase permeability, and cause leakage of intracellular constituents [37]. In particular, CAPE and galangin have been shown to destabilize lipid bilayers and exert strong antibacterial effects against Gram-positive bacteria [38]. However, while PI uptake supports a membrane-targeting component, this mechanism alone is unlikely to fully explain the antibacterial profile observed. Several phytochemicals detected in *Protegol*, particularly flavonoids and phenolic acids, are also known to interfere with bacterial energy metabolism, inhibit nucleic acid synthesis, or impair efflux pump function [39,40]. Essential oil constituents may additionally contribute to intracellular oxidative damage through ROS generation [41]. Therefore, the antibacterial activity of *Protegol* is likely the result of multiple interacting mechanisms, with membrane destabilization representing only one of several contributing pathways. The ability of *Protegol* to induce significant PI uptake at sub-inhibitory concentrations highlights the importance of membrane destabilization not only for direct bactericidal action but also for potentiating the effects of other constituents present in the formulation. Notably, the more marked susceptibility of *S. pyogenes* compared to *S. aureus* may reflect structural differences in the cell wall and membrane composition between streptococci and staphylococci. Streptococci possess a thinner peptidoglycan layer and lack certain protective enzymes, which may render them more sensitive to amphipathic phytochemicals capable of perturbing membrane stability [42]. These species-specific variations further underscore the broad but heterogeneous antibacterial profile of *Protegol*.

### 3.3. Anti-Inflammatory Activity of Protegol Against LPS-Induced Nitric Oxide Production

The results obtained in RAW 264.7 macrophages demonstrate that *Protegol* exerts a marked dose-dependent inhibition of nitric oxide (NO) production in response to LPS stimulation, with significant reductions already evident at 125 μg/mL and a strong suppression at higher concentrations. This effect suggests that *Protegol* can effectively modulate macrophage-mediated inflammatory responses, which play a pivotal role in the pathogenesis of respiratory tract infections. Excessive NO production is a hallmark of macrophage activation and contributes to tissue damage, oxidative stress, and exacerbation of inflammatory cascades during bacterial and viral infections of the airways [43]. The anti-inflammatory potential of *Protegol* can be attributed to the synergistic action of its main constituents. Propolis flavonoids, such as CAPE and galangin, are well known to inhibit inducible nitric oxide synthase (iNOS) expression and NF-κB activation in LPS-stimulated macrophages [44]. Similarly, honey polyphenols have been reported to attenuate pro-inflammatory cytokine release and reduce nitrite accumulation in vitro [45]. Among the essential oil components, 1,8-cineole, the major constituent of Eucalyptus globulus oil, is reported in the literature to exert potent anti-inflammatory activity by suppressing NF-κB signaling and reducing pro-inflammatory mediators in airway epithelial cells and macrophages [46]. Terpinen-4-ol, the main component of tea tree oil, also downregulates inflammatory mediator production in activated immune cells [47]. Taken together, these data suggest that the reduction in NO production by *Protegol* may result from the combined actions of phenolics, flavonoids, and terpenes, which are known to modulate inflammatory responses through multiple complementary mechanisms. Literature studies reported polyherbal formulations rich in natural extracts, endowed with significant anti-inflammatory activity in both cellular and animal models. However, these formulations are composed of different ingredients compared to *Protegol* and lack the antibacterial activity that characterizes *Protegol* [48]. Indeed, the ability of *Protegol* to attenuate NO production, together with its antimicrobial effects, highlights its potential as a natural adjuvant for managing airway inflammation. This property is of particular relevance in the context of respiratory tract infections, where exaggerated inflammatory responses contribute to disease severity, thus providing an additional value to the overall bioactivity profile described above. Although NO is a robust and widely used marker of macrophage activation, the LPS-induced inflammatory response also involves the production of key cytokines such as IL-1β, IL-6, and TNF-α, which orchestrate and amplify the pro-inflammatory cascade [49]. Several constituents of *Protegol*—such as 1,8-cineole, propolis-derived flavonoids, and honey polyphenols—have already been individually reported to modulate these mediators, further supporting the hypothesis that the formulation may exert broader anti-inflammatory effects beyond NO suppression [50,51,52]. The present findings therefore describe one of the major pathways involved in early macrophage activation, while additional cytokine networks not investigated here may also contribute to the overall anti-inflammatory profile of the formulation.

### 3.4. ROS Scavenging Effects of Protegol Against H_2_O_2_-Induced Oxidative Stress

The results of the DCFH-DA assay clearly demonstrate that *Protegol* exerts an appreciable dose-dependent antioxidant effect in BALB/3T3 fibroblasts exposed to oxidative stress induced by H_2_O_2_, with ROS levels significantly reduced with dose-dependant effects. These findings are noteworthy, given the well-known the role of oxidative stress in the pathophysiology of respiratory tract infections, where excessive ROS production contributes to tissue injury, immune dysregulation, and increased susceptibility to bacterial colonization [53]. The antioxidant activity of *Protegol* can be attributed to the combined action of its bioactive constituents. Propolis is particularly rich in flavonoids and phenolic acids such as caffeic acid, galangin, and chlorogenic acid, which are known for their strong free radical scavenging properties and ability to enhance endogenous antioxidant defenses [54]. Honey polyphenols also contribute to ROS attenuation by modulating redox-sensitive transcription factors and upregulating cellular antioxidant enzymes [55]. Essential oils included in the formulation provide additional antioxidant activity: 1,8-cineole has been reported to reduce ROS accumulation and protect cellular structures under oxidative stress conditions [35], while terpinen-4-ol and other terpenes from tea tree oil display significant ROS-scavenging and cytoprotective effects [56]. While DCFH-DA is a sensitive probe for detecting global intracellular ROS, it reflects only one dimension of the oxidative stress response. Several constituents of *Protegol* have been independently shown to modulate additional redox-regulating mechanisms—such as glutathione (GSH) homeostasis, lipid peroxidation, and antioxidant enzyme activity (SOD, CAT)—suggesting that the formulation may engage broader antioxidant pathways beyond those directly captured by the assay. For example, propolis flavonoids have been reported to increase intracellular GSH levels and activate SOD and catalase in oxidative stress models [57], honey polyphenols have been shown to support Nrf2-mediated cytoprotective responses [58], and 1,8-cineole has demonstrated the ability to reduce lipid peroxidation while restoring antioxidant enzyme activity [59]. These complementary activities provide biological context to the DCFH-DA findings and support the possibility that *Protegol* influences multiple levels of oxidative regulation. The additional strong reduction in ROS levels observed in fibroblasts highlights the relevance of *Protegol* as a natural strategy to counteract oxidative stress, an important pathogenic factor in respiratory infections and inflammatory airway diseases [60].

### 3.5. Comparison of Protegol with Other Similar Formulations

The search for multifunctional natural formulations targeting respiratory health has led to the development of different products combining propolis, honey, plant extracts, and essential oils. However, the composition, biological spectrum, and mechanistic characterization of these products vary significantly, making direct comparison challenging. In this context, a comparative analysis helps to highlight the specific features that distinguish *Protegol* from existing alternatives. Several illustrative examples are available. An oral/throat spray formulation based on propolis and essential oils demonstrated a broad range of bioactivities relevant to upper airway infections, including inhibition of *S. aureus* and *S. pyogenes*, reduction in pro-inflammatory cytokine expression, and attenuation of oxidative damage in epithelial models. In vivo, the same formulation showed protective effects on mucosal tissues, supporting its use as a prophylactic or early-intervention strategy for upper airway infections [28]. Another product—a dietary supplement combining *Pelargonium sidoides* extract, honey, propolis, and zinc—was evaluated in pediatric patients with acute tonsillopharyngitis and found to significantly accelerate the improvement of fever, odynophagia, nasal obstruction, and mucosal inflammation when used alongside standard therapy. Clinical assessments reported reduced symptom scores, earlier restoration of normal activity levels, and decreased need for escalation of therapy, highlighting the immunomodulatory and soothing potential of this multi-component preparation [29]. Furthermore, a randomized, double-blind, placebo-controlled clinical trial employing a standardized polyphenol mixture extracted from poplar-type propolis demonstrated faster remission of uncomplicated upper respiratory tract infection (URTI) symptoms, particularly sore throat, cough, and nasal congestion. The formulation also decreased the duration of systemic symptoms such as malaise and low-grade fever, suggesting combined antimicrobial, antiviral, and anti-inflammatory contributions to clinical improvement [30]. *Protegol* differs from these products for several distinguishing characteristics. First, its formulation integrates a broader and more functionally diverse set of bioactive components—including propolis, honey, Sisymbrium officinale, selected essential oils, vitamin C, and zinc—creating a complex multi-component supplement that acts simultaneously on microbial viability, mucosal protection, inflammatory pathways, and oxidative stress balance. While the compared formulations rely on two or few functional classes (propolis + essential oils, or propolis + honey + zinc), *Protegol* combines polyphenols, sulfur-containing plant constituents, essential oils, and micronutrients, potentially targeting multiple pathophysiological nodes relevant in respiratory infections. Second, the antibacterial spectrum of *Protegol* appears broader. Unlike many available natural formulations, whose clinical or preclinical data concern mainly Gram-positive pathogens, *Protegol* demonstrated in vitro activity against Gram-positive species (e.g., *Staphylococcus aureus*, *Streptococcus pyogenes*) and, notably, against multidrug-resistant Gram-negative pathogens (e.g., *Klebsiella pneumoniae*). This extended antibacterial profile, including membrane-disruptive effects, distinguishes *Protegol* from conventional propolis- or honey-based supplements that rarely show efficacy against Gram-negative or MDR strains. Finally, the experimental characterization provided for *Protegol* is more comprehensive than typically reported. While the above products have been studied essentially for clinical symptom relief, antimicrobial or anti-inflammatory effects, and *Protegol* has been evaluated for antibacterial activity (MIC/MBC), membrane permeability, anti-inflammatory (NO reduction), and antioxidant (ROS inhibition) properties within the same experimental framework. This multi-target and multi-endpoint approach better reflects the complex pathophysiology of respiratory tract infections, where microbial load, inflammation, and oxidative stress are interlinked, and supports the innovative value of *Protegol* among natural supplements for respiratory health.

## 4. Materials and Methods

### 4.1. Protegol

The *Protegol* supplement was kindly provided by Erbenobili S.r.l. (Corato, Italy). It consists of a hydroalcoholic formulation enriched with natural extracts and essential oils traditionally employed to support respiratory health. As stated by the manufacturer, the composition includes hydroalcoholic extracts (water, alcohol, plant part 20%) of propolis (from propolis resin) and hedge mustard (*Sisymbrium officinale* (L.) Scop.) aerial parts, together with honey. The composition is further supplemented with vitamin C (L-ascorbic acid) and zinc gluconate, two micronutrients recognized for their immunomodulatory and antioxidant properties. In addition, *Protegol* includes a blend of essential oils derived from manuka (*Leptospermum scoparium* J.R. Forst. & Forst.) leaves, eucalyptus (*Eucalyptus globulus* Labill.) leaves, and tea tree (*Melaleuca alternifolia* (Maiden & Betche) Cheel) leaves, which are widely documented for their antimicrobial and anti-inflammatory activities.

### 4.2. Chemicals

The following reagents were obtained from Sigma-Aldrich S.p.A. (Milan, Italy): 3-(4,5-dimethylthiazol-2-yl)-2,5-diphenyltetrazolium bromide (MTT), lipopolysaccharide (LPS, *Escherichia coli* 0111:B4), Griess reagent, and 2′,7′-dichlorofluorescein diacetate (DCFH-DA). Propidium iodide (PI) for membrane permeability evaluation was also supplied by Sigma-Aldrich, whereas hydrogen peroxide (H_2_O_2_) served as a positive control in oxidative stress assays. Cell culture reagents included high-glucose (4.5 g/L) Dulbecco’s Modified Eagle Medium (DMEM), fetal bovine serum (FBS; Euroclone S.p.A., Pero, Italy), L-glutamine, and trypsin-EDTA solution. All additional chemicals were of analytical grade and obtained from standard commercial suppliers.

### 4.3. Chemical Characterization

#### 4.3.1. Sample Preparation

To 3.5 mL of *Protegol*, 10.5 mL of hexane were added, resulting in the formation of two distinct phases. The upper organic phase (OP) was evaporated to dryness and re-dissolved in hexane to obtain a solution at a concentration of 3 mg/mL, which was stored for subsequent gas chromatography analysis. The lower phase (polar fraction) was also evaporated to dryness and re-dissolved in methanol to yield a solution at a concentration of 50 mg/mL, which was then analyzed by HPLC-DAD.

#### 4.3.2. Organic Phase

A Trace GC–FID Ultra gas chromatograph (Thermo Finnigan, Bremen, Germany) was employed for chemical analysis. The organic phase (OP) was dissolved in hexane prior to analysis, and 1 μL of the solution was injected. A fused silica capillary column (30 m × 0.25 mm i.d.; 0.25 μm film thickness) coated with DB-5 stationary phase (J&W Scientific, Milan, Italy) was used for cold on-column injection. The chromatographic conditions were as follows: the detector temperature was set at 300 °C, and the column oven temperature was programmed from 60 °C (isothermal for 5 min) to 280 °C (isothermal for 15 min) at a rate of 4 °C/min. Hydrogen was used as the carrier gas at a pressure of 35 kPa and a flow rate of 2.0 mL/min. Data were processed using the Chrom-Card 32-bit version 2.0 computing software. The composition of OP components was calculated based on the relative area of the peaks in the GC-FID chromatograms, without the application of correction factors. Gas chromatography–mass spectrometry (GC–MS) analysis was performed using a Hewlett Packard 6890 gas chromatograph coupled with a 5973 mass spectrometer, controlled by an HP ChemStation system (Agilent Technologies, Palo Alto, CA, USA). The GC conditions included an injector temperature of 280 °C and an oven temperature program from 60 °C (isothermal for 5 min) to 270 °C (isothermal for 30 min), with a ramp rate of 4 °C/min. Helium was used as the carrier gas at a flow rate of 1 mL/min. The capillary column used was HP-5 MS (30 m × 0.25 mm i.d.; 0.25 μm film thickness). The mass spectrometer operated under the following conditions: vacuum pressure of 10^−5^ Torr, ion source temperature of 200 °C, and electron current of 34.6 μA. Mass spectra were recorded at a scan rate of 1 scan per second over a mass range of 40–800 amu. The ionization mode used was electron impact (EI). Samples (1 μL) were injected in splitless mode. The chemical composition of the OP was identified by comparing the GC retention times of the analytes with those of authentic reference standards (purchased from Sigma-Aldrich, Milan, Italy), supported by Kovats Retention Index (KI) values and mass spectral data from both standard compounds and the NIST mass spectral library [61].

#### 4.3.3. Polar Fraction

##### HPLC-DAD Analyses

Phenolic acids and flavonoids from polar fraction were quantitatively analyzed using an Agilent Technologies 1260 Infinity HPLC system equipped with a photodiode array (PDA) detector and a Gemini C18 column (250 × 4.6 mm, 5 μm particle size; Phenomenex, Torrance, CA, USA). The mobile phases consisted of water containing 0.1% formic acid (solvent A) and acetonitrile containing 0.1% formic acid (solvent B). The elution gradient was set as follows: starting at 10% B, increasing to 40% B at 40 min, and reaching 60% B at 60 min. The flow rate was 1 mL/min, and the injection volume was 25 μL. UV spectra were recorded at 210, 280, 310, and 350 nm. All analyses were performed in triplicate. Quantification of phenolic acids was carried out using HPLC/PDA based on a calibration curve generated with caffeic acid (Sigma-Aldrich) across eight concentrations within the linear range of 31.25–500 μg/mL. The resulting calibration curve showed excellent linearity, with a correlation coefficient (R^2^) of 0.9992 and the equation y = 67.475x + 1395.2. Quantification of flavonoids was performed using a calibration curve established with Kaempferol-3-*O*-glucoside (Extrasynthese) at five concentrations within the linear range of 62.5–1000μg/mL. The standard curve also demonstrated high linearity, with a correlation coefficient (R^2^) of 0.9991 and the equation y = 62.774x + 747.63.

##### ESI-MS/MS Analyses

Compound identification was carried out using an Agilent 1100 Series LC/MSD Trap VL system. Data acquisition and processing were performed with Agilent ChemStation software (LC/MSD Trap Software 4.1; Agilent Technologies, Santa Clara, CA, USA, 2002). Analyses employed an electrospray ionization (ESI) source operating in both positive and negative ionization modes. The ESI parameters were set as follows: capillary voltage, 4000 V; nebulizer gas (N_2_) pressure, 15 psi; drying gas (N_2_) temperature, 350 °C; and flow rate, 5 L/min. Full-scan mass spectra were acquired over a mass-to-charge (m/z) range of 100–2200, with a scan speed of 13,000 m/z/s. Automated ESI-MS/MS experiments were performed by isolating the molecular ion (base peak) with an isolation width of 4.0 m/z, a threshold of 100, and ion charge control enabled, with a maximum acquisition time of 300 ms. MS/MS fragmentation was carried out using conventional collision energies of 1.0, 10.0, and 30.0 V. The sample was prepared in methanol at concentrations of 20–30 ppm and introduced into the system at a flow rate of 10 μL/min using a KD Scientific Syringe Pump (KD Scientific Inc., Holliston, MA, USA).

### 4.4. Antibacterial Studies

The antibacterial properties of *Protegol* were evaluated by means of the broth microdilution procedure in accordance with the Clinical and Laboratory Standards Institute (CLSI) guidelines (M07-A9, 2012) [62], with levofloxacin serving as a reference antibiotic. Both reference and clinical bacterial strains were included in the study. The Gram-positive panel comprised *Staphylococcus aureus* ATCC 25923, ATCC 29213, ATCC 43300 (methicillin-resistant), and a clinical isolate (*S. aureus* BS, obtained from the Civil Hospital “Casa della Divina Provvidenza”, Bisceglie, Bari, Italy), along with Streptococcus pyogenes ATCC 19615 and its clinical isolate (*S. pyogenes* F), *S. mutans* ATCC 25175, *S. pneumoniae* ATCC 10015, *S. salivarius* ATCC 13419, and *S. sanguinis* ATCC 10556. The Gram-negative strains included *Klebsiella pneumoniae* ATCC 13883, ATCC 700603, and a clinical isolate (*K. pneumoniae* BS), as well as *Pseudomonas aeruginosa* ATCC 27853. Clinical isolates were recovered from positive blood cultures of hospitalized patients and kindly provided by the Hygiene Section, Department of Biomedical Sciences and Human Oncology, University of Bari (Italy). Strains were identified through standard physiological and morphological procedures using API identification systems (API 20S, API Rapid Staph, API Rapid 20E; bioMérieux SA, Marcy-l’Étoile, France). As expected, the clinical isolates of *S. aureus*, *S. pyogenes*, and *K. pneumoniae* exhibited multidrug resistance to several antibiotic classes. Reference strains from the American Type Culture Collection (ATCC) were used as quality controls with susceptibility ranges established by CLSI. *Protegol* was dissolved and serially diluted in cation-adjusted Mueller–Hinton broth (CAMHB). Levofloxacin, used as positive control, was dissolved in DMSO (20 mg/mL) and further diluted in CAMHB to obtain the required concentrations. Each bacterial strain was grown in Mueller–Hinton broth at 37 °C for 3–5 h, and the turbidity was adjusted to the 0.5 McFarland standard (OD625 = 0.08–0.10). The cultures were then diluted 1:100 to obtain a final inoculum of approximately 1–2 × 10^6^ CFU/mL, and 200 μL of each suspension were distributed into 96-well microplates. Following 24 h of incubation at 37 °C, the minimum inhibitory concentration (MIC) was defined as the lowest concentration that completely inhibited visible bacterial growth. For minimum bactericidal concentration (MBC) evaluation, 10 μL from clear wells were plated onto Mueller–Hinton agar and incubated for an additional 24 h. MBC was defined as the lowest concentration causing ≥99.9% reduction in viable cells. All assays were conducted in triplicate and repeated independently three times to ensure reproducibility.

### 4.5. Cell Wall Permeability Assay

The impact of *Protegol* on bacterial membrane integrity was evaluated through the propidium iodide (PI) uptake assay, as previously described with minor modifications [63]. PI is a membrane-impermeant dye that penetrates only damaged cells, binding to nucleic acids and producing a fluorescent signal proportional to the loss of membrane integrity. Based on the results of MIC/MBC determination, two strains were selected for this assay due to their higher susceptibility to *Protegol*: *Staphylococcus aureus* ATCC 29213 and the clinical isolate *Streptococcus pyogenes* F. *Staphylococcus aureus* ATCC 29213 and the clinical isolate *Streptococcus pyogenes* F, were grown to mid-logarithmic phase and then treated with *Protegol* at MIC, ½ MIC, and ¼ MIC values for 2 h at 37 °C. Propidium iodide (PI), dissolved in phosphate-buffered saline (PBS, pH 7.4), was added to bacterial suspensions at a final concentration of 10 μg/mL and incubated for 30 min at 37 °C. Subsequently, 100 μL aliquots of each sample were transferred to black 96-well microplates, and fluorescence intensity was recorded using a microplate reader (excitation 535 nm, emission 615 nm; Tecan Infinite M1000 Pro, Tecan, Cernusco S.N., Italy). Results were expressed as mean ± standard deviation (SD) of at least three independent experiments performed in triplicate.

### 4.6. Cell Culture

Murine macrophage RAW 264.7 and mouse fibroblast BALB/3T3 cell lines were purchased from the American Type Culture Collection (ATCC, Manassas, VA, USA) and maintained at 37 °C in a humidified incubator (95% air, 5% CO_2_). Cells were grown in high-glucose Dulbecco’s Modified Eagle Medium (DMEM; Euroclone S.p.A., Pero, Italy) supplemented with 10% fetal bovine serum (FBS), 1% L-glutamine, and 100 U/mL penicillin–streptomycin. The culture medium was replaced every 2–3 days, and subculturing was performed at 80–90% confluence. All reagents used for cell culture were of analytical grade.

### 4.7. Evaluation of Cytotoxicity Using MTT Assay

The MTT (3-(4,5-dimethylthiazol-2-yl)-2,5-diphenyltetrazolium bromide) assay was used to investigate the potential cytotoxicity of *Protegol*. RAW 264.7 macrophages and BALB/3T3 fibroblasts were seeded into 96-well plates at a density of 5 × 10^3^ cells/well and allowed to adhere overnight. The cells were then treated with increasing concentrations of *Protegol* (125, 250, and 500 μg/mL) for 24 h. After treatment, 20 μL of MTT solution (5 mg/mL in PBS) was added to each well and incubated for 3 h at 37 °C. The resulting formazan crystals were solubilized with 100 μL of DMSO, and absorbance was measured at 570 nm using a microplate reader (Infinite M1000 Pro, Tecan, Cernusco S.N., Italy). Each condition was analyzed in triplicate and repeated in at least three independent assays. Cell viability was expressed as a percentage relative to the untreated control [64].

### 4.8. Measurement of Intracellular ROS Production

Intracellular reactive oxygen species (ROS) levels were determined in BALB/3T3 cells exposed to oxidative stress using the 2′,7′-dichlorofluorescein diacetate (DCFH-DA) assay, following a slightly modified literature protocol [65]. Cells were seeded in black 96-well plates (5 × 10^3^ cells/well) and allowed to adhere overnight. After attachment, they were incubated with *Protegol* at 125, 250, or 500 μg/mL for 1 h, then challenged with 50 μM H_2_O_2_ for 30 min to induce oxidative stress. During the last 30 min of incubation, cells were exposed to DCFH-DA (25 μM final concentration). Subsequently, cells were washed twice with PBS to remove unbound dye, and fluorescence was recorded with a microplate reader (excitation 485 nm, emission 535 nm; Infinite M1000 Pro, Tecan, Cernusco S.N., Italy). Each condition was tested in triplicate and repeated in at least three independent assays. Fluorescence values were normalized to untreated controls and expressed as a percentage of ROS production relative to the H_2_O_2_-stimulated group [66].

### 4.9. Evaluation of Anti-Inflammatory Activity Using the Griess Assay

The anti-inflammatory activity of *Protegol* was evaluated by quantifying nitric oxide (NO) release in LPS-stimulated RAW 264.7 macrophages using the Griess colorimetric assay. Cells were seeded in 96-well plates (1 × 10^5^ cells/well) and incubated for 24 h at 37 °C in a humidified atmosphere containing 5% CO_2_. After adherence, cells were pre-exposed to *Protegol* at 125, 250, and 500 μg/mL for 1 h, followed by stimulation with lipopolysaccharide (LPS, 1 μg/mL) to trigger NO production. After 24 h of incubation, equal volumes (100 μL) of culture supernatant and Griess reagent (Sigma-Aldrich, Milan, Italy) were mixed according to the manufacturer’s instructions, incubated for 10 min at room temperature, and read at 540 nm using a microplate reader (Infinite M1000 Pro, Tecan, Italy). Nitrite levels, reflecting NO production, were quantified using a sodium nitrite calibration curve. All treatments were conducted in triplicate and replicated in at least three independent experiments.

### 4.10. Statistical Analysis

All results are expressed as mean ± standard deviation (SD) and obtained from at least three independent experiments. Each assay was conducted in triplicate to guarantee reproducibility. Statistical evaluation was carried out using one-way ANOVA followed by Dunnett’s post hoc test in GraphPad Prism 9.0, with significance thresholds set at *p* ≤ 0.05. Highly significant differences (*p* < 0.0001) are specifically indicated.

## 5. Conclusions

The present work explored the biological activities of the polyherbal formulation *Protegol*, emphasizing its antibacterial, anti-inflammatory, and antioxidant potential in in vitro models that mimic key aspects of respiratory infections. In antibacterial assays, *Protegol* exerted bactericidal effects, particularly against Gram-positive respiratory pathogens such as *Staphylococcus aureus* and *Streptococcus pyogenes*, with MIC values as low as 1.5 mg/mL and MBC values of 3.1 mg/mL. Notably, activity was also observed against multidrug-resistant Gram-negative bacteria, including *Klebsiella pneumoniae*, with MIC values up to 6.2 mg/mL and MBC values of 12.4 mg/mL, which is remarkable for a natural formulation, considering that many natural products, and even several synthetic agents, show no activity against these highly resistant strains. Propidium iodide uptake experiments further indicated that disruption of membrane integrity could represent a possible mechanism of the antimicrobial activity, with PI fluorescence increasing to 163% in *S. aureus* and 272% in *S. pyogenes* F at MIC concentrations and still elevated at sub-MIC levels (150% and 124% in *S. aureus* at ½ and ¼ MIC; 251% and 200% in *S. pyogenes* F at ½ and ¼ MIC). Additionally, *Protegol* exhibited dose-dependent antioxidant effects in BALB/3T3 fibroblasts exposed to oxidative stress, reducing ROS accumulation from 82% at 125 μg/mL to 45% at 250 μg/mL and 24% at 500 μg/mL. Similarly, in LPS-stimulated RAW 264.7 macrophages, the formulation significantly reduced nitric oxide production, decreasing levels to 82% at 125 μg/mL, 64% at 250 μg/mL, and 45% at 500 μg/mL. Overall, these findings describe a set of in vitro biological activities that reflect the multifaceted composition of *Protegol*. The observed effects likely arise from the concerted actions of multiple phytochemicals. However, the precise molecular pathways involved remain to be fully elucidated. Although the present data are limited to in vitro models, they provide a robust proof-of-concept and a solid foundation for further investigation. Future studies, including in vivo models and clinical research, will be essential to verify whether these in vitro effects translate into physiological relevance, to characterize the pharmacokinetic behavior of the formulation, and to determine its possible contribution as a complementary strategy in the context of respiratory tract conditions.

## Figures and Tables

**Table 1:** Minimum inhibitory concentration (MIC, mg/mL) and minimum bactericidal concentration (MBC, mg/mL) of *Protegol*.

	*Protegol*	
Gram-Positive Strains	MIC (mg/mL)	MBC (mg/mL)
*Staphylococcus aureus* ATCC 25923	1.5	3.1
*Staphylococcus aureus* ATCC 29213	1.5	3.1
*Staphylococcus aureus* ATCC 43300 (MRSA)	3.1	6.2
*Streptococcus mutans* ATCC 25175	3.1	6.2
*Streptococcus pneumoniae* ATCC 10015	6.2	12.4
*Streptococcus pyogenes* ATCC 19615	3.1	6.2
*Streptococcus salivarius* ATCC 13419	6.2	12.4
*Streptococcus sanguinis* ATCC 10556	3.1	6.2
Gram-Negative Strains		
*Klebsiella pneumoniae* ATCC 13883	3.1	6.2
*Klebsiella pneumoniae* ATCC 700603	6.2	12.4
*P. aeruginosa* ATCC 27853	6.2	12.4
Clinical Isolates		
*Staphylococcus aureus* BS	3.1	3.1
*Streptococcus pyogenes* F	1.5	3.1
*Klebsiella pneumoniae* BS	6.2	12.4

## Data Availability

The original contributions presented in this study are included in the article.

## Supplementary Materials

The following supporting information can be downloaded at: https://www.mdpi.com/article/10.3390/antibiotics14121260/s1, Table S1: Protegol polar fraction composition; Table S2: Constituents (%) of the organic phase from Protegol as determined by GC–FID and GC–MS analyses.
