# Supplementary material for: Exploring the Antibacterial, Anti-Inflammatory, and Antioxidant Properties of the Natural Food Supplement “Protegol” as a Supportive Strategy in Respiratory Tract Infections
Source: Antibiotics (Basel). 2025 Dec 13;14(12):1260. doi: 10.3390/antibiotics14121260 (PMC12730059; doi:10.3390/antibiotics14121260)
Supplement: Supplementary file 1 [file antibiotics-14-01260-s001.zip › antibiotics-3985808-supplementary.pdf]

Table S1. Protegol polar fraction composition.

Compound	*µg/ml ± SD	UV λ_{max} (nm)	$[M-H]^-$	ESI-MS/MS (%)
**Caffeic acid**	0.79 ± 0.01	298, 322	179	135(100)
**p-Coumaric acid**	0.50±0.06	298, 308	163	145(100)
**Ferulic acid**	0.18±0.01	294, 327	193	175(75), 143(12)
**Isoferulic acid**	0.37±0.01	294, 322	193	175(60), 143(35)
**3,4- dimethyl- caffeic acid (DMCA)**	1.27±0.02	296, 320	207	189(80), 161(40), 131(27)
**Pinobanksin 5-methylether**	1.62±0.05	286, 318sh	285	267(25), 239(13)
**Quercetin 3-methyl-ether**	0.38±0.01	254, 288sh, 356	315	300(48), 271(4)
**Pinobanksin**	2.04±0.02	290, 318sh	271	253(37), 225(12)
**Apigenin**	0.27±0.01	266,334	269	251(11)
**Kaempferol**	0.31±0.02	264,364	271	253(45)
**Isorhamnetin**	0.40±0.02	254, 370	315	300(48), 283(24)
**Quercetin dimethyl-ether**	0.25±0.07	254, 302sh, 354	329	314(11), 299(100), 285(4),243(6)
**Galangin 5-methyl-ether**	0.20±0.02	280, 298sh, 350	283	239(12), 179(8), 161(26), 133(49)
**Quercetin 7-methyl-ether**	0.76±0.03	254, 302sh, 354	315	300(48), 243(20)
**Caffeic acid benzyl ester**	0.95±0.02	298, 324	269	161(13),133(100)
**Caffeic acid prenyl ester**	2.19±0.06	298, 326	247	179(5), 161(13),135(86)
**Chrysin**	6.71±0.11	268, 312sh	253	151(21)
**Pinocembrin**	4.22±0.11	288, 330sh	255	213(64)
**Caffeic acid phenylethyl ester (CAPE)**	3.19±0.06	298, 322	283	179(8), 161(26),135(100)

Table S2. Constituents (%) of the organic phase from *Protegol* as determined by GC–FID and GC–MS analyses.

**Compound**	***KI tab.**	***KI cal.**	%
β-pinene	978	980	0.03
1,8-cineol	1033	1033	38. 81
Limonene	1031	1073	0.08
γ-terpinene	1062	1070	1. 46
Linalol	1098	1138	0.09
Canphora	1143	1165	0.14
terpinen-4-ol	1177	1178	21. 36

2-undecanone	1291	1287	0. 59
Cyclosativene	1368	1365	0.67
(z)-*β*- damascone	1383	1383	0.45
β -longipinene	1398	1395	0.30
(z)-caryophyllene	1404	1403	1. 24
β -gurjunene	1432	1434	0. 79
α- guaiene	1439	1439	0. 52
Khusimene	1447	1450	0.16
α-humelene	1454	1456	0.08
9-EPI-(e)-caryophyllene	1467	1470	1. 22
γ-gurjunene	1473	1477	2. 54
γ-muurolene	1477	1479	2. 81
β -selinene	1485	1488	4. 26
trans-β -guaiene	1500	1501	1. 13
6-methyl-alpha-(e)ionone	1518	1510	0.45
cis-calamenene	1521	1520	6. 77
Δ-cadinene	1524	1527	0.71
α-calacorene	1542	1535	1. 81
spathulenol	1576	1576	0.60
Gleenol	1585	1587	0.67
Cedrol	1596	1596	1. 17
trans-arteannuic alcohol	1607	1607	0.64
5-cedranone	1618	1618	1. 06
γ-eudesmol	1630	1630	1. 42
β-acorenal	1634	1633	0.53
Cubenol	1642	1642	3. 52
α-muurolol	1645	1649	0.28
selin-11-en-4-alpha-ol	1652	1665	0.64
n-heptadecane	1700	1699	0.37
1-octadecene	1793	1795	0.65

Data are expressed as mean of three repetitions ± SD (n = 3).MS fragmentation and UV-vis absorption data of compounds detected in the polar fraction of *Protegol* by HPLC-DAD (280,310 and 350 nm)
